# Lupeol acetate as a potent antifungal compound against opportunistic human and phytopathogenic mold *Macrophominaphaseolina*

**DOI:** 10.1038/s41598-021-87725-7

**Published:** 2021-04-19

**Authors:** Shabnam Javed, Zaid Mahmood, Khalid Mohammed Khan, Satyajit D. Sarker, Arshad Javaid, Iqra Haider Khan, Amna Shoaib

**Affiliations:** 1 Institute of Chemistry, University of the Punjab, Quaid-e-Azam Campus, Lahore, Pakistan; 2 H. E. J. Research Institute of Chemistry, International Centre for Chemical and Biological Sciences, University of Karachi, Karachi, Pakistan; 3 Centre for Natural Products Discovery, School of Pharmacy and Biomolecular Sciences, Liverpool John Moores University, James Parsons Building, Byrom Street, Liverpool, L3 3AF UK; 4 Department of Chemistry and Environmental Sciences, University of Lahore, Lahore, Pakistan; 5 Institute of Agricultural Sciences, University of the Punjab, Quaid-e-Azam Campus, Lahore, Pakistan

**Keywords:** Plant sciences, Chemistry

## Abstract

Antifungal activity of *Monotheca* *buxifolia* methanolic extract and its various fractions were assessed against *Macrophomina* *phaseolina,* a soil-borne fungal pathogen of more than 500 vegetal species as well as rare and emerging opportunistic human pathogen. Different concentrations of methanolic extract (3.125 to 200 mg mL^−1^) inhibited fungal biomass by 39–45%. Isolated *n-*hexane, chloroform and ethyl acetate fractions suppressed fungal biomass by 32–52%, 29–50% and 29–35%, respectively. Triterpenes lupeol and lupeol acetate **(1**, **2**) were isolated from n-hexane while betulin, β-sitosterol, β-amyrin, oleanolic acid (**3–6**) were isolated from chloroform fraction. Vanillic acid, protocatechuic acid**,** kaempferol and quercetin **(7–10)** were isolated from the ethyl acetate fraction and identified using various spectroscopic techniques namely mass spectroscopy and NMR. Antifungal activity of different concentrations (0.0312 to 2 mg mL^−1^) of the isolated compounds was evaluated and compared with the activity of a broad spectrum fungicide mancozeb. Different concentrations of mencozeb reduced fungal biomass by 83–85%. Among the isolated compounds lupeol acetate **(2**) was found the highest antifungal against *M.* *phaseolina* followed by betulin **(3)**, vanillic acid (**7**), protocatechuic acid **(8),** β-amyrin (**5**) and oleanolic acid **(6)** resulting in 79–81%, 77–79%, 74–79%, 67–72%, 68–71% and 68–71%, respectively. Rest of the compounds also showed considerable antifungal activity and reduced *M.* *phaseolina* biomass by 41–64%.

## Introduction

*Macrophomina* *phaseolina* is an important soil-borne plant pathogen that causes diseases over 500 plant species including economically important crops such as legumes, sunflower, cotton, sorghum and vegetables. Generally it causes charcoal rot disease in various crops; however, it also causes other diseases such as seedling and stem blight, damping off and wilt^1^. It has vast distribution in tropical and subtropical countries, however, its exposure to human may cause infection in immunosuppressed patients. Several strategies are being adopted to control fungal plant pathogens by synthetic fungicides. No doubt, these fungicides are effective in controlling plant diseases but they also pose severe hazards to human health and cause environmental pollution by accumulation in soil and water^2^. It necessitates alternative environmental friendly strategies for management of phytopathogens. Many recent studies have shown that crude plant extracts as well as purified compounds isolated from various plant species are very effective in the control of fungal plant pathogens^3–5^. Studies have shown that plant extracts of *Chenopodium* spp., *Senna* *occidentalis* and *Cirsium* *arvense* can control growth of *M.* *phaseolina* and charcoal rot of mungbean^6–10^. From Azadirachta and Mango leaves, three flavonoids (–)-epi-catechin, (−)-epicatechin-3-*O*-β-glucopyranoside and 6-(phydroxybenzyl)taxifolin-7-*O*-β-d-glucoside were isolated and found effective against *M.* *phaseolina*^11,12^.

*Monotheca* *buxifolia* (Falc.) A. DC. is monotypic genus of the family Sapotacea, grows mainly in Malakand Dir district, Pakistan. *M.* *buxifolia* is reported for wide-range of pharmacological activities including cough, wound healing, headache, analgesic, antipyretic, antiseptic and urinary tract infections^13^. The antibacterial activity of crude ethanolic extract and fractions of *M.* *buxifolia* using agar well diffusion technique confirmed their antibacterial potential^14^. Javed et al.^15^ isolated and identified five triterpenes from the bioactive fractions of aerial parts of *M.* *buxifolia* and displayed potent cytotoxic activities in vitro. The other plants of family Sapotaceae are widely explored and reported for significant antimycotic activities in various experimental models, but there is not only a single report on antifungal activity and isolation of antifungal compounds from *M.* *buxifolia*^16^. Therefore, the present investigation was conducted to explore antifungal potential of *M.* *buxifolia* fractions and isolated compounds against *M.* *phaseolina,* a highly problematic phytopathogen for which there is not any registered fungicide up to now.

## Results

### Characteristic of the compounds

#### Compound 1

EI-MS *m/z* (relative intensity): 426(52) [M]^+^, 411(20), 393 (8), 218(42), 207(80), 189(100), 139(74). EIMS showed fragment peaks at *m/z* 411 [M^+^-CH_3_], 218 [M^+^-C_14_H_20_], 207(80) which are characteristic signals for pentacyclic triterpenes having isopropenyl group. HREI-MS *m/z*: 426.3820 (calcd. for C_30_H_50_O, 426.3862). The ^1^H-NMR spectrum of 24 showed seven singlet for methyl protons at *δ* 0.74, 0.81, 0.85, 0.89, 0.91, 0.97 and 1.06 signals, one for each of seven methyl protons. A pair of multiplets at *δ* 4.20 and 4.21, each for H-29, identified the presence of terminal isopropenyl group, characteristic of lupane series of triterpenoids. ^13^C NMR spectrum showed 30 signals for the triterpenoid lupane skeleton, including C-3 at *δ* 79.2 bonded to the hydroxyl group. Compound **1** was identified as Lupeol^19^.

#### Compound 2

HREI-MS showed [M]^+^ 468.3967 correlated with C_32_H_52_O_2 ._ EI-MS showed fragments 427 [M^+^—41], 408 [M^+^-AcOH], 249 [M^+^-C_16_H_27_] and 189 [M^+^-C_16_H_27_-AcOH]. ^1^H-NMR spectrum of **2** recorded seven tertiary methyl group at *δ* 0.76, 0.81, 0.83, 0.86, 0.92, 1.01, 1.31 and methyl acetate at 2.32. A pair of broad singlets at *δ* 4.47 and 4.44 showed presence of exomethylene group. A double doublet at *δ* 4.03, *J* = 9.8, 4.3 Hz was due to C-3 proton, bonded to acetoxy group. The coupling constant along with chemical shift describes the absolute configuration of acetoxy group, β and equatorial at C-3. The recorded data of **2** is comparable with the data reported for lupeol acetate in the literature^20^.

#### Compound 3

HR-EI-MS spectrum showed [M]^+^ at 442.3819 deduced for molecular formula C_30_H_50_O_2_ depicted six degrees of unsaturation. ^1^H-NMR spectrum showed *δ* at 3.25 (*dd*, *J,* 11.4, 5.2 Hz) was attributed due to H-3 germinal to ^_^OH proton. Two broad singlets appeared at *δ* 4.46 and 4.92 (*br* *s,* 1H each) were assigned to terminal isopropenyl group. Six tertiary methyl singlets were observed at *δ* (0.86, 0.83, 0.84, 0.87, 1.02, 1.04) were assumed for methyl protons of (H-23, H-24, H-25, H-26, H-27 and H-30). One doublet appeared at *δ* 3.40 and 3.71 (2H, *d*, *J* = 11.2 Hz) was due to methylene protons of C-28. Compound **3** can be identified as Betulin^21^.

#### Compound 4

HR-EI-MS spectrum showed [M]^+^ at 414.3838, correlated with molecular formula C_29_H_50_O (calcd. *m/z*: 414.3661). Characetristic fragment ions *m/z* 414 and 396 in EI-MS spectrum also confirmed β-sitosterol pattern. Similarly fragment ion *m/z* 255 was recorded due to loss of [M^+^-side chain-H_2_O] and fragments *m/z*: 329, 303 confirmed the diagnostic pattern for sterols with ∆^5^-unsaturation. The ^1^H-NMR spectrum also confirmed the characteristic steroid pattern, one triplet at *δ* 5.41 (*J* = 3.2 Hz) was assigned for olefinic proton of C-6 and a carbinylic signal was recorded at *δ* 3.45 (1H, *m*). Some doublet signals appeared at *δ* 0.902 (*J* = 6.4), 0.86 (*J* = 6.6), 0.82 (*J* = 6.6), 0.89 (*J* = 6.8) and were recommended for C-21, 26, 27 and C-29 methyl protons. The ^13^C-NMR spectrum of compound **4** showed 29 carbons with 6 methyl, 11 methylene, 9 methine and 3 quaternary carbon atoms. The compound **4** was identified as β-sitosterol, also confirmed by reported data in literature^22^.

#### Compound 5

HR-EI-MS showed M]^+^ at *m/z* 426.3534, established molecular formula C_30_H_50_O, calculated by 426.3860. Eight tertiary methyl protons were identified in the ^1^H NMR spectrum (500 MHz, CD_3_OD) *δ* 0.80, 0.84, 0.88, 0.95, 1.12, 1.34, 1.01 and 1.11, each (3H, *s*). C-12 olefinic proton was also indicated at *δ* 5.22 (1H, *br*, *s*) and a double doublet at *δ* 3.61 (1H, *dd*, *J* = 8, 4.4 Hz) was due to C-3 proton. The ^13^C NMR (BB and DEPT) spectrum showed presence of 8 –CH_3_, 10 –CH_2_ , 5 –CH and 7-quaternary carbons. The spectroscopic data was identical with the data of well-known compound reported in the literature, commonly known as β-amyrin^23^.

#### Compound 6

HR-EI-MS spectrum showed the [M]^+^ at *m/z* 456. 3671 recommended for C_30_H_48_O_3_ calculated for 456.3603. EI-MS spectrum in addition to recording molecular ion peak *m/z* 456 showed characteristic fragment ions *m/z* 248, 203 and 133 for ∆^12^ amyrin skeleton. The ^1^H NMR spectrum displayed the signal for olefininc proton at *δ* 5.18 (1H, *br* *s*, H-12) and oxymethine proton germinal to hydroxyl group at *δ* 3.14 (1H, *dd*, *J* = 10, 5.5 Hz). Seven singlets were also showed for seven methyl group protons were recorded at *δ* (1.19, 1.02, 0.92, 0.88, 0.80, 0.74 and 0.71). ^13^C NMR spectrum showed 30 carbon signals comprising of 7 –CH_3_, 10 –CH_2_, 5 –CH and 8 –C carbon atoms. Carbonyl carbon showed prominent signal at *δ* 180.8*,* olefinic carbons *δ* 124.4 and oxymethine carbon *δ* 78.2. The above spectral data for compound **6** was in complete agreement Oleanolic acid^24^.

#### Compound 7

HREI-MS spectrum showed [M]^+^ at 168.1124 (calculated 168.1120) for C_8_H_8_O_4_. In EI-MS spectra base peak [M]^+^ is represented at 168 and other peaks at 151 and 137 represent the loss of –OH and –OCH_3_ groups. ^1^H-NMR spectrum (500 MHz, CD_3_OD) of compound **7** presented a doublets at *δ* 6.80 (*J* = 8.2) and a *dd* at 7.55 with (*J* = 8.2 and 1.6 Hz) were assigned C-5 and C-6 protons. One proton doublet at *δ* 7.60 with (*J* = 1.6 Hz), was assigned as C-2 proton. Singlet, three proton signal at *δ* 3.88 was attributed due to three protons of methoxy group. Another singlet at *δ* 4.59 was assigned to hydrogen of hydroxyl group. Compound **7** was thus identified as (4-hydroxy-3,5-dimethoxy benzoic acid) commonly known as vanillic acid. The ^1^H and ^13^C NMR spectroscopic data was also verified with the previous reported data for vanillic acid^25^.

#### Compound 8

^1^H-NMR spectrum of compound **8** showed presence of three aromatic hydrogens, which gives information that three positions of benzene ring might be substituted. Compound **8** gave one double doublet at *δ* 7.45 with (*J* = 8.2, 1.8 Hz) was assigned to C-6 proton due to possibility of *ortho* coupling with C-5 proton and *meta* coupling with C-2 proton. Two doublets appeared for single protons in aromatic region can be assigned to C-5 proton with (*J* = 8.2 Hz) due to *ortho* coupling with C-6 proton and to C-2 proton with (*J* = 1.8 Hz) due to *meta* coupling with C-6 proton respectively. H-decoupled ^13^C-NMR spectrum, seven peaks appeared for six non-equivalent carbons and one carboxylic carbon. The calculated mass for compound 18 with molecular formula C_7_H_6_O_4_ was 154.2338 and 154.2331 was observed again confirms the compound as protocatechuic acid^26^.

#### Compound 9

Molecular ion peak [M]^+^ was recorded at 286.0475 which is compatible for molecular formula C_15_H_10_O_6_. EI-MS showed characteristic fragments at 257, 229, 213, 121,104, 93, 77 and 69. ^1^H NMR spectrum displayed two doublets at *δ* 8.03 and *δ* 6.92 representing AA'BB' pattern for *para*-substituted benzene. Two *meta* coupled doublets also appeared at *δ* 6.42 and 6.18 with *J* = 1.8 Hz. Characteristic flavonoid signals 137.2, 146.2 were shown clearly in ^13^C NMR (DEPT and BB) spectra. ^13^C NMR signals at *δ* 161.4, 160.2 and 114.2 represent the *para*-substituted benzene ring moiety. Similarly carbon atoms directly linked with oxygen displayed signals at 161.4, 103.4 and 160.2. The spectroscopic data was also matched with the reported data in literature and compound (**9**) was identified as 3,4′,5,7-tetrahydroxyflavone commonly known as kaempferol^27^.

#### Compound 10

Compound (**10**) HR-EI-MS spectra depicted molecular ion peak at *m/z* 302.0285 with relevant molecular formula C_15_H_10_O_6_. The ^1^H-NMR spectrum of (**10**) showed five signals each for five different aromatic protons. A doublet signal at 7.72 represents single proton in *meta* coupling with H-6′, 2.1 Hz might be attributed due to H-2′. A double doublet at 7.62 represents H-6′, in *meta* coupling with H-2′, *J* = 2.1 Hz and *ortho* coupling with H-5′, *J* = 8.4 Hz. Another doublet at 6.87 might be due to H-5′, *J* = 8.4. It suggested an ABC type-system having flavonol moiety with 3′, 4′-disubstituted B-ring. Similarly a pair of *meta* coupling protons signals at 6.17 (1H, *d*, *J* = 1.8, H-6) and 6.38 (1H, *d*, *J* = 1.8, H-8) were recorded for ring A. ^13^C-NMR spectra showed 15 signals having carbonyl signal at δ 170.5 for C-4. Five carbon nuclei attached to hydroxyl group gave five signals recorded at δC 137.1 (C-3), 160.9 (C-5), 165.1 (C-7), 146.4 (C-3′), 150.8 (C-4′) also supported the presence of 3,5,7,3′,4′-oxygenated flavone nucleus. The detailed studies on compound **10** were in consistent with the reported spectroscopic data, commonly known as quercetin^28^.

### Antifungal activity of fractions and isolated compounds

**Figure 1:**
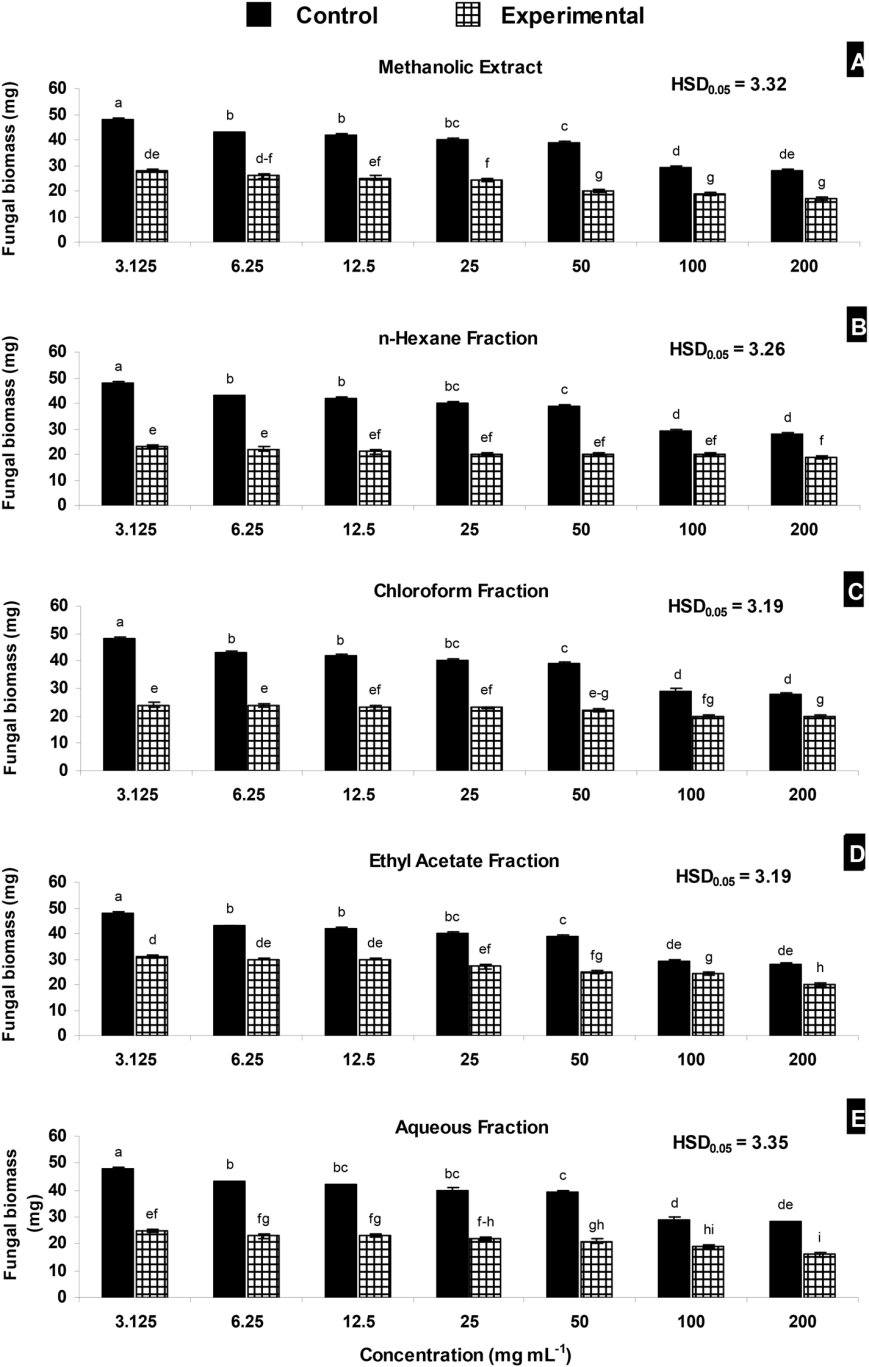
**(A–E)** Effect of methanolic extract of *Monotheca* *buxifolia* and its fractions on biomass of *Macrophomina* *phaseolina*. Vertical bars show standard errors of means of three replicates. Values with different letters at their top show significant difference (P ≤ 0.05) as determined by Tukey’s HSD test.

Different concentrations of methanolic extract suppressed fungal biomass by 34–49%. Likewise, there was 31–52%, 29–50%, 17–36% and 34–48% reduction in biomass of *M.* *phaseolina* due to different concentrations of methanolic *n-*hexane, chloroform, ethyl acetate and aqueous fraction of methanolic leaf extract, respectively (Fig. 1).

Antifungal activity of mencozeb and the ten isolated compounds against *M.* *phaseolina* is shown in Table 1. Fungicide mencozeb showed the highest antifungal activity causing 83–84% reduction in fungal biomass. In general, all the isolated compounds suppressed fungal growth variably and significantly. Out of the isolated compounds, lupeol acetate (**2**) showed the highest antifungal against *M.* *phaseolina*, causing 79–81% followed by betulin (**3**) 77–79% reduction in biomas of the target fungus, respectively. Other compounds showing pronounced antifungal activities were β-amyrin (**5**), oleanolic acid (**6**)**,** vanillic acid (**7**) and protocatechuic acid (**8**) that reduced fungal biomass by 68–71%, 68–71%, 74–79% and 67–72%, respectively. Compounds namely lupeol (**1**), β-sitosterol (**4**), kaempferol (**9**) and quercetin (**10**) were comparatively less antifungal and reduced fungal biomass by 40–43%, 57–64%, 46–53% and 47–55%, respectively over corresponding control.

**Table 1:** Effect of different concentrations of fungicide mancozeb and compounds isolated from *Monotheca* *buxifolia* on growth appearance and biomass of *Macrophomina* *phaseolina.*

Methanolic extract fraction	Conc. of DMSO (µL mL^−1^)	Compound conc (mg mL^−1^)	Fungal biomass (mg)	Decrease over control (%)
Control	0.104	0.0000	23.4 A	–
	0.208	0.0000	22.6 AB	–
	0.416	0.0000	22.8 AB	–
	0.832	0.0000	21.1 BC	–
	1.66	0.0000	21.3 B	–
	3.33	0.0000	21.0 BC	–
	6.66	0.0000	19.2 C	–
Mencozeb	0.104	0.0312	3.9 Za–e	83
	0.208	0.0625	3.7 a–e	84
	0.416	0.1250	3.8 a–e	83
	0.832	0.2500	3.5 d–e	83
	1.66	0.5000	3.5 b–e	83
	3.33	1.0000	3.3 c–e	85
	6.66	2.0000	3.1 e	84
Lupeol **(1)**	0.104	0.0312	13.3 DE	43
	0.208	0.0625	13.4 D	41
	0.416	0.1250	13.4 D	41
	0.832	0.2500	12.8 D–F	39
	1.66	0.5000	12.3 D–G	42
	3.33	1.0000	12.5 D–G	40
	6.66	2.0000	11.6 D–H	40
Lupeol acetate **(2**)	0.104	0.0312	4.6 T-Za–e	80
	0.208	0.0625	4.6 T-Za–e	80
	0.416	0.1250	4.3 V-Za–e	81
	0.832	0.2500	4.3 V-Za–e	80
	1.66	0.5000	4.0 Y-Za–e	81
	3.33	1.0000	3.9 Za–e	81
	6.66	2.0000	4.0 Y–Za–e	79
Betulin **(3)**	0.104	0.0312	5.7 O–Za	76
	0.208	0.0625	5.3 P–Za–c	77
	0.416	0.1250	5.2 P–Za–d	77
	0.832	0.2500	4.5 U–Za–e	79
	1.66	0.5000	4.7 S–Za–e	78
	3.33	1.0000	4.5 U–Za–e	79
	6.66	2.0000	4.2 X–Za–e	78
*β*-Sitosterol **(4**)	0.104	0.0312	9.4 I–K	60
	0.208	0.0625	9.4 I–K	58
	0.416	0.1250	9.0 J–L	61
	0.832	0.2500	9.1 J–L	57
	1.66	0.5000	8.0 K–M	62
	3.33	1.0000	7.7 K–N	64
	6.66	2.0000	7.7 K–N	60
*β*-Amyrin (**5**)	0.104	0.0312	7.4 L–O	68
	0.208	0.0625	6.9 M–Q	69
	0.416	0.1250	6.5 M–U	71
	0.832	0.2500	6.6 M–T	69
	1.66	0.5000	6.6 M–T	69
	3.33	1.0000	6.2 M–X	70
	6.66	2.0000	6.2 M–X	68
Oleanolic acid **(6)**	0.104	0.0312	6.8 M–R	71
	0.208	0.0625	6.7 M–S	70
	0.416	0.1250	6.7 M–S	71
	0.832	0.2500	6.3 M–V	70
	1.66	0.5000	6.5 M–U	69
	3.33	1.0000	6.3 M–V	70
	6.66	2.0000	6.0 N–Y	68
Vanillic acid **(7)**	0.104	0.0312	6.2 M–X	74
	0.208	0.0625	5.6 O–Za	76
	0.416	0.1250	5.6 O–Za	79
	0.832	0.2500	5.4 O–Zab	74
	1.66	0.5000	4.7 S–Za–e	78
	3.33	1.0000	4.9 R–Za–e	77
	6.66	2.0000	4.9 R–Za–e	74
Protocatechuic acid **(8)**	0.104	0.0312	6.9 M–Q	71
	0.208	0.0625	6.7 M–S	70
	0.416	0.1250	6.9 M–Q	70
	0.832	0.2500	6.9 M–Q	67
	1.66	0.5000	6.5 M–U	72
	3.33	1.0000	6.2 M–X	70
	6.66	2.0000	5.9 N–Z	69
Kaempferol **(9)**	0.104	0.0312	12.3 D–G	50
	0.208	0.0625	11.6 D–H	50
	0.416	0.1250	11.3 E–I	50
	0.832	0.2500	11.3 E–I	46
	1.66	0.5000	10.8 G–J	58
	3.33	1.0000	9.0 J–L	57
	6.66	2.0000	9.1 J–L	53
Quercetin **(10)**	0.104	0.0312	12.0 D–H	49
	0.208	0.0625	12.0 D–H	47
	0.416	0.1250	11.8 D–H	48
	0.832	0.2500	11.2 F–I	47
	1.66	0.5000	10.1 H–J	53
	3.33	1.0000	9.5 I–K	55
	6.66	2.0000	9.0 J–L	53
	HSD_0.001_		2.01	

Values with different letters in a column show significant difference (P ≤ 0.001) as determined by Tukey’s HSD test.

## Discussion

### Identification of isolated compounds

**Figure 2:**
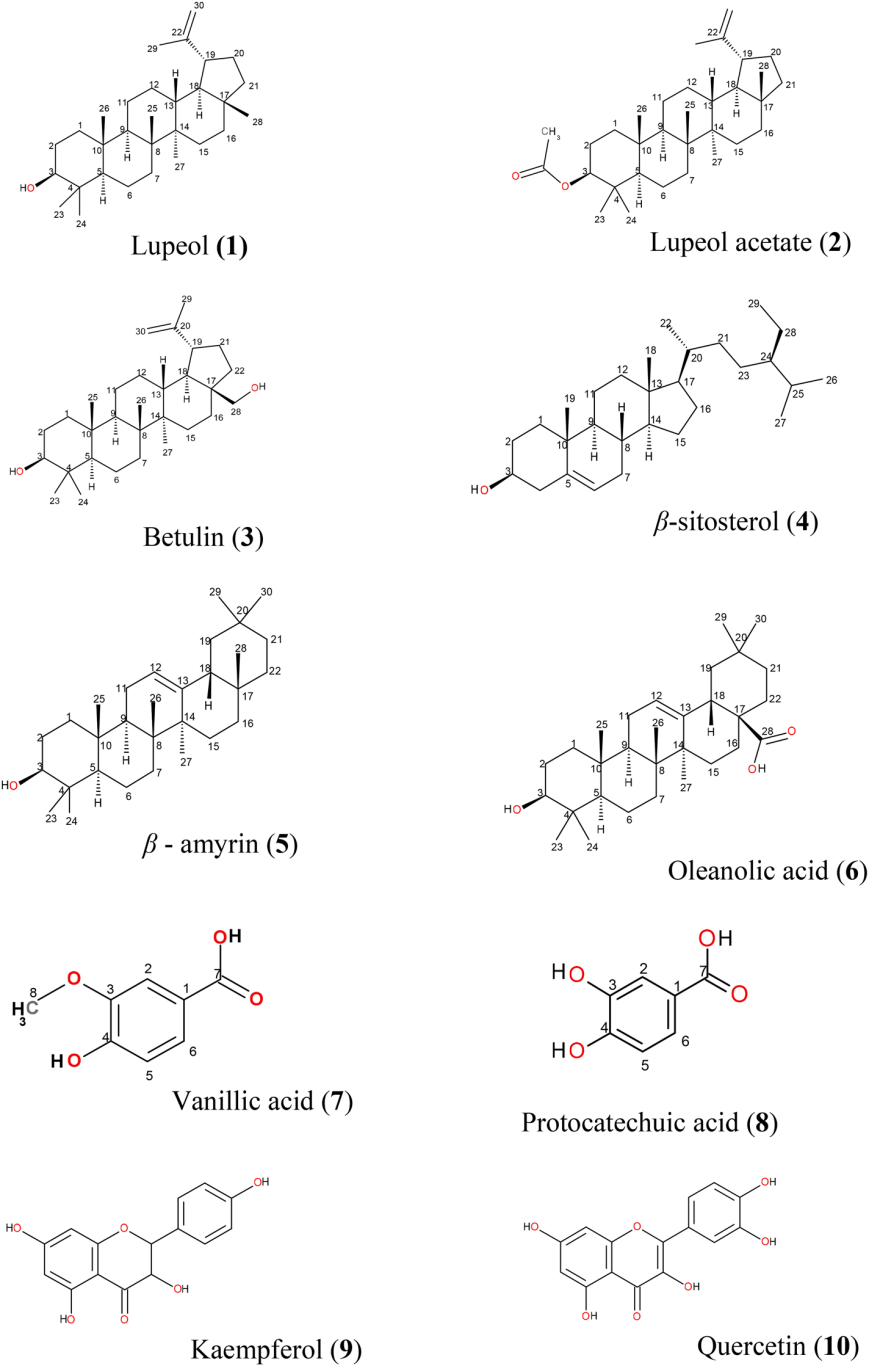
Structures of compounds isolated from *n-*hexane (**1** and **2**), chloroform (**3–6**) and ethyl acetate (**7–10**) fraction of methanolic extract of *Monotheca* *buxifolia*.

From *M.* *buxifolia* methanolic extract ten compounds were isolated from various sub-fractions. Various physical analysis and spectroscopic data co-ordinated from the already reported data and compound **1** was identified as lupeol, compound **2** as lupeol acetate, compound **3** as betulin, compound **4** as β-sitosterol, compound **5** as β-amyrin, compound **6** as oleanolic acid, compound **7** as vanillic acid, compound **8** as protocatechuic acid, compound **9** as kaempferol and compound **10** as quercetin^19–28^ Structures of these compounds are presented in Fig. 2.

### Antifungal potential of fractions and isolated compounds

All the concentrations of methanolic extracts and its various fractions significantly reduced fungal biomass. Antifungal activity of both compounds lupeol acetate (**2**) 79–81% and betulin (**3**) 77–79% were found very close to that of fungicide mancozeb 83–84%. Some earlier studies also reported antifungal activity of lupeol acetate (**2**) against *Aspergillus* *flavus.* However, most of the previous studies showed antibacterial activity of this compound against *Bacillus* *subtilis*, *Staphylococcus* *aureus* and *Escherichia* *coli*^29,30^. In contrast to the present study, Manzano et al.^31^ reported no antifungal activity of **2** against *Penicillium* *chrysogenum* and *Fusarium* *oxysporum*, showing specificity of this compound against *M.* *phaseolina.* Betulin (**3**) has been reported fungicidal against a number of fungi including *Fusarium* *solani* and *Aspergillus* *niger* and its effect was equal to standard antifungal drug miconazole^32^.

β-Amyrin (**5**) is known to inhibit growth of various clinical fungal species namely *Candida* *stellatoidea,* *Candida* *krusei,* *Microsporum* sp. and *Trichophyton* *rubrum* and *Ascochyta* *rabiei,* the cause of blight disease of chickpea^33,34^ Oleanolic acid (**6**) is a pentacyclic triterpenoid that is widely spread in plant kingdom, with members of Oleaceae family as its main source. Apart from its various pharmaceutical properties, it also possesses antifungal activity against yeast and dermatophyte species^35,36^. Protocatechuic acid (**8**) is a type of phenolic acid that is found in many food plants and possesses a number of pharmaceutical properties and antifungal activity against *Microsporum* *audouinii*^37,38^.

Lupeol (**1**), a pentacyclic triterpenoid, has also been isolated from a number of plant species including green pepper, mangoes, strawberry, grapes, white cabbage and olive, and is known to have useful effect as a preventive and therapeutic agent against a number of ailments^39^. Although in the present study, it found comparatively less effective against *M.* *phaseolina,* however, it was highly effective against *Penicillium* *notatum* causing 90% growth inhibition when used in 200 µg mL^−1^ concentration^31^. It was also inhibitory to the growth of *Fusarium* *solani,* *Aspergillus* *niger,* *Rhizoctoia* *phaseoli,* *Candida* *albicans,* *Penicillium* *chrysogenum,* *Cantharellus* *flavus* and *Microsporum* *canis,* and its antifungal activity was comparable with miconazole^40^. *β*-Sitosterol (**4**) is known to inhibit growth of *Aspergillus* *niger* and *Cladosporium* *cladosporioides* at a concentration of 0.01 mg mL^−1^. It also inhibited growth of germ tube of *Fusarium* *verticillioides* by 82% at 50 mg mL^−1^^41^. Quercetin (**10**) is a polyphenolic flavonoid and showed a low activity against different *Candida* species^42^. This compound is also known to increase efficacy of a fungicidal compound amphotericin B against a clinical fungal species *Cryptococcus* *neoformans*^43^. Kaempferol (**9**) is a phenolic compounds generally showing antibacterial activity^44^. Information regarding its antifungal activity are very rare. The present study concludes that aerial parts of *M.* *buxifolia* contain potentent antifungal constituents especially lupeol acetate **(2**) causing 79–81% reduction followed by betulin (**3**) 77–79% and vanillic acid (**7**) 74–79% for the management of *M.* *phaseolina,* a highly problematic phytopathogen for which there is not any registered fungicides so far.

## Materials and methods

### Chemicals and reagents

Chemicals were attained from Sigma-Aldrich Germany. For isolation work including chromatography various solvents were acquired from Fischer Scientific (Loughborough, UK). Silica and Flash column silica (70–230 mesh) and (230–400 mesh) were assimilated from E. Merck, Darmstadt, Germany.

### Physical parameters and spectroscopic analysis

Melting point apparatus Buchi 535 was used to record melting point. Digital Polarimeter; Jasco DIP-360 was used to record optical rotation. Shimadzu UV-240 spectrophotometer was used to record λ_max_ in nm. Infrared spectra (IR) were recorded on Shimadzu IR-460 in nujol or KBr pellets and reported in cm^−1^. NMR spectra were recorded on Bruker AMX-500 and AM-400 for^1^H (300–500 MHz) and^13^C (125–150 MHz) spectrometers. "spectra were recorded on Bruker Avance spectrometers ranging from 7.05 up to 14.09 T". Mass spectrometer Jeol-JMS H X-110 was employed to record high resolution electron ionization mass spectra (HR-EIMS).

### Collection, extraction and fractionation from plant material

*M.* *buxifolia* is wild and commonly grown plant species in mountains of Swat, Pakistan. Aerial parts viz., stem and leaves (11 kg) of *M.* *buxifolia* were collected from Swat, Pakistan according to prescribed rules in The Pakistan Trade Control of Wild Fauna and Flora Act, 2012. Species was identified by Dr. Arshad Javaid (Associate Professor, Institute of Agricultural Sciences, University of the Punjab, Lahore, Pakistan), assigned voucher no. GC. Bot. Herb. 815 (*Monotheca* *buxifolia*) and was deposited in the Dr. Sultan Ahmed Herbarium, Department of Botany, GC University, Lahore, Pakistan.

Aerial parts were dried under shade, pulverized and weighed (4.5 kg). Powdered plant material was soaked in MeOH 95% (16 L × 4). Extracts were assembled and methanol was removed under reduced pressure at 50 °C temperature. Gummy mass (586 g) having dark brown colour was collected. This MeOH extract was added in water (410 mL) and further fractionation was done using *n-*hexane, chloroform and ethyl acetate (10 × 3 L) each to afford 151, 102 and 152 g of the fractions respectively. Aqueous fraction was collected 161 g and all the portions were refrigerated^17^.

### Isolation of *Macrophomina phaseolina*

Diseased root pieces of mungbean were collected, washed and cut into small pieces of 0.5 cm. Root pieces were surface sterilized using 0.1% sodium hypochlorite solution, transferred aseptically on autoclaved malt extract agar plates and incubated for 7 days at 27 °C. Grey fungal colonies appeared on root pieces which became darken with time. The colonies were further purified by growing on fresh malt extract agar plates. The fungus was isolated and identified as *M.* *phaseolina* (FCBP-0751) on the basis of characteristic black-colored oblong microsclerotia^18^.

### Antifungal bioassays with *M. buxifolia* extract/fractions

The methanolic extract and its four fractions were tested against the pathogenic fungus *M.* *phaseolina* in vitro. An amount of 1.2 g of methanolic extract and its various fractions was dissolved in 1 mL of dimethyl sulphoxide (DMSO) and added to 5 mL autoclaved malt extract broth to make stock solution of 200 mg mL^−1^ concentration. Six lower concentrations viz. 100, 50, 25, 12.5, 6.25 and 3.125 mg mL^−1^ were prepared from stock solution in series of double dilution. Similarly, control with respect to each concentration was prepared by dissolving 1 mL DMSO in 5 mL malt extract broth and double diluted in series. Experiment was performed in glass test tubes (10 mL), each containing 1 mL of growth medium. Tubes were inoculated aseptically with 20 µL of *M.* *phaseolina* suspension and each treatment was replicated three times. After 7 days of incubation at room temperature, fungal biomass was filtered, dried and weighed.

### Isolation and purification of compounds

*M.* *buxifolia* lipophilic hexane fraction was subjected to vacuum liquid chromatography over silica gel and eluted with increasing order of solvent polarity as hexane–EtOAc (0 → 10). Hexane first sub-fraction, collected using *n*-hexane: EtOAc (7:3) was again chromatographed to isolate compound 1 (10 mg) using same polarity system. Hexane second sub-fraction collected using *n*-hexane–EtOAc (6:4) was again chromatographed to isolate compound 2 (8 mg) eluted with same solvent system. CHCl_3_ fraction was subjected to silica gel column and eluted with *n*-hexane-chloroform (10:0 → 0:0) to chloroform–methanol (0:0 → 0:10). Chloroform fraction lead to isolation of three main sub-fractions. CHCl_3_ sub-fraction 1 was further purified and compound 3 and 4 (13,10 mg) were obtained with *n*-hexane-CHCl_3_ (7:3) *n*-hexane-dichloromethane (5:5) and (6:4) as eluent, respectively. Sub-fraction 2 eluted with *n*-hexane-chloroform (6:4) was purified with *n*-hexane:dichloromethane (5:5) to obtain compound 5 (15 mg). CHCl_3_ sub-fraction 3 eluted with chloroform–methanol (9.5:0.5) was rechromatographed using dichloromethane-methanol (9:1) to afford compound 6 (30 mg). The EtOAc fraction was chromatographed using silica gel and eluted with solvent system of increasing polarity *n*-hexane, *n*-hexane: DCM and DCM:MeOH and three sub-fractions were collected. EtOAc sub-fraction 1, eluted by *n*-hexane: DCM (3:7) was refilled on silica gel and eluted by *n-*hexane:DCM (1:9) to attain compound 4 (4 mg) and 7 (6 mg). EtOAc sub-fraction 2 eluted by DCM 100%, was reloaded and eluted by same solvent system to separate compound 8 (8 mg). EtOAc sub-fraction 3 was isolated using DCM:MeOH (9:1) was rechromatographed and eluted with identical solvent system to collect compound 9 and 10 (6,8 mg).

### Physical properties, spectral analysis of pure compounds

#### Lupeol (1)

Melting point, 215 °C was recorded. ^1^H-NMR (400 MHz, CD_3_OD), *δ* ppm: 4.20 and 4.21 (2H, br*,* 1H each, H-29), 3.30 (1H, *dd*, *J* = 9.4, 4.6 Hz, H-3), 0.74, 0.81, 0.85, 0.89, 0.91, 0.97, 1.06 (3H, 7 s, 7 Me). ^13^C-NMR (150 MHz, CD3OD) *δ* ppm: 150.7 (C-20), 109.7 (C-29), 79.2 (C-3), 55.2 (C-5), 50.3 (C-9), 48.3 (C-18), 47.8 (C-19), 43.2 (C-17), 42.7 (C-14), 40.7 (C-8), 39.9 (C-22), 38.6 (C-4), 38.2 (C-1), 38.0 (C-13), 37.3 (C-10), 35.9 (C-16), 35.0 (C-7), 29.7 (C-21), 28.4 (C-22), 28.2 (C-23)27.9 (C-2), 27.2 (C-15), 25.3 (C-12), 21.1 (C-11), 19.3 (C-30), 18.4 (C-6), 18.2(C-28), 15.8 (C-25), 15.5 (C-26), 15.3 (C-27), 15.1 (C-24). EI-MS *m/z* [M]^+^ at : 426.

#### Lupeol acetate (2)

IR spectrum showed absorption at 1730 cm^−1^. ^1^H-NMR (500 MHz, CD_3_OD), *δ* ppm: 4.47 and 4.44 (2H, br*,* 1H each, H-29), 4.03 (1H, *dd*, *J* = 9.8, 4.3 Hz, H-3), 0.76, 0.81, 0.83, 0.86, 0.92, 1.01, 1.31 (3H, 7 s, 7 Me) and 2.32 (3H,*s*, CH_3_COO). ^13^C- NMR (150 MHz, CDCl_3_) *δ* ppm: 172.2 (C-1′), 150.2 (C-20), 110.5 (C-29), 81.1 (C-3), 50.7 (C-9), 48.7 (C-18), 48.4 (C-19), 38.5 (C-1), 22.7 (C-2), 37.2 (C-4), 55.3 (C-5), 18.3 (C-6), 34.8 (C-7), 41.2 (C-8), 40.1 (C-22), 37.2 (C-10), 21.2 (C-11), 24.6 (C-12), 37.2 (C-13), 43.0 (C-14), 26.4 (C-15), 35.8 (C-16), 43.1 (C-17), 29.9 (C-21), 28.2 (C-23), 20.4 (C-2′), 19.3 (C-30),16.1 (C-24), 16.3 (C-25), 15.4 (C-26), 14.2 (C-27), 14.8 (C-28). EI-MS *m/z* [M]^+^ at : 468.

#### Betulin (3)

Compound **3** showed IR spectrum at 3457 cm^−1^. ^1^H-NMR (500 MHz, CDCl_3_), *δ* ppm: 4.46 and 4.92 (*br* *s,* 1H each, H-29), 3.40 and 3.71 (2H, *d*, *J* = 11.2 Hz, H-28), 3.25 (*dd*, *J* = 11.4, 5.2 Hz, H-3), 0.83 (H_3_-24), 0.84 (H_3_-25), 0.86 (H_3_-23), 0.87 (H_3_-26), 1.02 (H_3_-27), 1.04 (H_3_-30) (3H, 6 s, 6 Me). ^13^C- NMR (150 MHz, CDCl_3_) *δ* ppm: 150.8 (C-20), 109.8 (C-29), 79.1 (C-3), 60.4 (C-28), 38.4 (C-1), 55.3 (C-5), 50.6 (C-9), 48.6 (C-19), 46.6 (C-17), 42.5 (C-18), 42.8 (C-14), 27.5 (C-2), 39.2 (C-4), 37.5 (C-13), 36.2 (C-22), 30.2 (C-21), 18.4 (C-6), 34.3 (C-7), 39.5 (C-8), 37.2 (C-10), 21.0 (C-11), 24.2 (C- 12), 27.2 (C-15), 30.3 (C-16), 28.1 (C-23), 19.1 (C-30), 16.1 (C-25), 16.2 (C-26), 15.2 (C-24), 14.8 (C-27). EI-MS *m/z* [M]^+^ at : 442.

#### β-sitosterol (4)

^1^H-NMR (500 MHz, CDCl_3_), *δ* ppm: 5.41 (1H,*t*, *J* = 3.2 Hz, H-6), 3.45 (1H, *m*, H-3), 1.034 (3H, *s,* Me-19), 0.902 (3H*,d,J* = 6.4, Me-21), 0.89 (3H *,d,J* = 6.8, Me-29), 0.86 (3H*,d,* *J* = 6.6, Me-26), 0.82 (3H*,d,J* = 6.6, Me-27), 0.68 (3H*,s,* Me-18). ^13^C-NMR (150 MHz, CDCl_3_) *δ* ppm: 141.2 (C-5), 122.1 (C-6), 72.2 (C-3), 56.8 (C-14), 56.2 (C-17), 51.9 (C-9), 46.4 (C-24), 42.4 (C-4), 41.8 (C-13), 40.6 (C-12), 36.8 (C-1), 35.8 (C-10), 34.4 (C-20), 32.2 (C-22), 31.8 (C-7), 31.6 (C-8), 31.2 (C-2), 30.4 (C-25), 28.6 (C-16), 25.4 (C-23); 24.8 (C-15), 23.8 (C-28), 22.8 (C-11), 21.2 (C-26), 20.6 (C-19), 19.8 (C-27), 18.9 (C-21), 12.8 (C-29), 12.6 (C-18). EI-MS *m/z* [M]^+^ at : 414.

#### β-amyrin (5)

Absence of absorption in UV region was indicated. ^1^H-NMR (500 MHz, CD_3_OD), *δ* ppm: 5.22 (1H, *br* *s*, H-12), 3.61 (1H, *dd*, *J* = 8, 4.4 Hz, H-3), 0.80 (3H, *s*), 0.84 (3H, *s*), 0.88 (3H, *s*), 0.95 (3H*,* *s*), 1.12 (3H, *s*), 1.34 (3H, *s*) , 1.01 (3H, *s*) and 1.11 (3H, *s*). ^13^C-NMR (150 MHz, CDCl_3_) *δ* ppm: 145.6 (C-13), 121.4 (C-12), 78.8 (C-3), 47.2 (C-18), 46.6 (C-19), 37.8 (C-1), 28.1 (C-2), 38.4 (C-4), 55.6 (C-5), 18.8 (C-6), 33.1 (C-7), 38.2 (C-8), 47.8 (C-9), 37.2 (C-10), 23.4 (C-11), 41.2 (C-14), 26.8 (C-15), 27.3 (C-16), 32.2 (C-17), 31.1 (C-20); 34.2 (C-21), 37.3 (C-22), 28.9 (C-23), 15.5 (C-24), 15.6 (C-25), 16.7 (C-26), 26.4 (C-27), 28.8 (C-28), 33.4 (C-29), 23.8 (C-30). EI-MS *m/z* [M]^+^ at : 426.

#### Oleanolic acid (6)

IR spectrum showed absorption band 3430–2650, 1680 and 1650–850 cm^-^ respectively.^1^H-NMR (500 MHz, CDCl_3_), *δ* ppm: 5.18 (1H, *br* *s*, H-12), 3.14 (1H, *dd*, *J* = 10, 5.5 Hz, H-3), 1.19 (3H, *s*), 1.02 (3H, *s*), 0.92 (3H, *s*), 0.88 (3H*,* *s*), 0.80 (3H, *s*), 0.74 (3H, *s*) and 0.71 (3H, *s*). ^13^C-NMR (150 MHz, CDCl_3_) *δ* ppm: 180.8 (C-28), 145.8 (C-13), 124.4 (C-12), 78.2 (C-3), 55.9 (C-5), 48.5 (C-9), 47.2 (C-17), 46.6 (C-19), 42.5 (C-18), 42.4 (C-14), 39.4 (C-4), 38.8 (C-1), 33.8 (C-29), 33.3 (C-7), 40.1 (C-8), 37.1 (C-10), 31.4 (C-20); 34.8 (C-21), 33.1 (C-22), 29.8 (C-23), 24.1 (C-11), 28.8 (C-15), 28.2 (C-2), 26.8 (C-27), 24.2 (C-16), 23.6 (C-30), 19.1 (C-6), 17.2 (C-26), 16.4 (C-24), 16.1 (C-25). EI-MS *m/z* [M]^+^ at : 456.

#### Vanillic acid (7)

The UV λ_max_ showed absorption at 282 nm. The infrared spectrum displayed absorption bands at 3400–2700 and 1670 cm^−1^ indicated the presence of conjugated carboxylic acid. ^1^H-NMR (500 MHz, CD_3_OD), *δ* ppm: 7.60 (1H, *d*, 1.6 Hz, H-2), 7.55 (1H, *dd*, *J* = 8.2, 1.6 Hz, H-6), 6.80 (1H, *d*, *J* = 8.2, H-5), 4.59 (1H,*s*, OH), 3.88 (3H, *s*, OCH_3_). ^13^C- NMR (150 MHz, CD_3_OD) *δ* ppm: 55.9 (CH_3_O-3), 112.3 (C-2), 114.3 (C-5), 121.9 (C-6), 124.0 (C-1), 146.6 (C-3), 150.5 (C-4). EI-MS *m/z* [M]^+^ at : 168.

#### Protocatechuic acid (8)

UV absorption was recorded at 274 nm, IR spectrum displayed absorption bands at 3210–2610 cm^*−*1^. ^1^H-NMR (500 MHz, CD_3_OD), *δ* ppm: 7.45 (1H, *dd*, *J* = 8.2, 1.8 Hz, H-6), 7.42 (1H, *d*, *J* = 1.8 Hz, H-2), 6.82 (1H, *d*, *J* = 8.2 Hz, H-5), ^13^C- NMR (150 MHz, CDCl_3_) *δ* ppm: 123.4 (C-1), 120.9 (C-2), 147.2 (C-3), 148.9 (C-4), 117.4 (C-5), 118.5 (C-6), 170.5 (C-7). EI-MS *m/z* [M]^+^ at : 154.

#### Kaempferol (9)

Melting point at 276–278 °C was recorded. UV spectrum recorded λ_max_ at 265, 212 and 336 (4.0) nm while IR spectrum showed absorption at 3412, 2926, 1660, 1610, 1600–1525 and 1380 cm^*−*1^. ^1^H-NMR (400 MHz, DMSO), *δ* ppm: 8.03 (2H, *d*, *J* = 8.8 Hz, H-2′, -6′), 6.92 (2H, *d*, *J* = 8.8 Hz, H-3′,-5′), 6.42 (1H, *d*, *J* = 1.8 Hz, H-8), 6.18 (1H, *d*, *J* = 1.8 Hz, H-6).^13^C- NMR (150 MHz, CD3OD) *δ* ppm: 178.1 (C-4), 161.4 (C-5), 160.2 (C-7), 159.4 (C-4′), 155.9 (C-9), 146.2 (C-2), 137.2 (C-3), 128.3 (C-2′, -6′), 121.3 (C-1′), 114.2 (C-3′, -5′), 103.4 (C-10), 99.8 (C-6), 93.4 (C-8). EI-MS *m/z* [M]^+^ at : 286.

#### Quercetin (10)

Melting point was recorded at 314–316 °C. Two characteristic absorption bands in UV spectrum at 250 and 360 nm were showed. HR-EI-MS spectra depicted molecular ion peak at *m/z* 302.0285 with relevant molecular formula C_15_H_10_O_6_. IR spectrum showed two characteristic bands at 3402 and 1610 cm^−1^. ^1^H-NMR (300 MHz, CD_3_OD), *δ* ppm: 7.72 (1H, *d*, 2.1 Hz, H-2′), 7.62 (1H, *dd*, *J* = 8.4, 2.1 Hz, H-6′), 6.87 (1H, *d*, *J* = 8.4, H-5′), 6.38 (1H, *d*, *J* = 1.8, H-8), 6.17 (1H, *d*, *J* = 1.8, H-6). ^13^C-NMR (150 MHz, CD3OD) *δ* ppm: 170.5 (C-4), 165.1 (C-7), 160.9 (C-5), 160.0 (C-9), 150.8 (C-4′), 146.4 (C-3′),146.2 (C-2), 137.1 (C-3), 124.2 (C-1′), 121.4 (C-6′)117.0 (C-2′,-5′), 104.2 (C-10), 99.2 (C-6), 94.1 (C-8). EI-MS *m/z* [M]^+^ at : 302.

### Antifungal bioassays with pure compounds from *M. buxifolia*

Two compounds **(1, 2)** were isolated from *n*-hexane fraction, four compounds **(3–6)** from chloroform fraction and four compounds **(7–10)** from ethyl acetate fraction of the methanolic extract of *M.* *buxifolia* were tested for MIC values by microdilution assay. MIC values of mancozeb as reference synthetic fungicide (80%WP, KSS) and isolated compounds were tested by serial dilution in culture tubes. Six milligrams of each of the ten isolated compounds and mancozeb (active ingredient) were dissolved in 20 µL DMSO and added to autoclaved malt extract to raise the volume up to 3 mL, to prepare a growth medium of 2 mg mL^−1^ concentration. Further serial double dilutions viz. 1, 0.5, 0.25…0.0312 mg mL^−1^ were made using malt extract broth in culture tubes. Spore suspension was prepared by adding 10 day old fungal culture in double distilled water. DMSO (20 µL mL) was added in malt extract broth to prepare 3 mL of control that serially double diluted to make corresponding control treatments for each concentration. Each treatment was replicated thrice. Fugal suspension (20 µL) was added to each concentration (0.5 mL) of the growth medium and incubated at 27 °C. Fungal biomass was collected on filter papers after 3 days growth, dried and weighed.

### Statistical analysis

All the data were analyzed by analysis of variance followed by Tukey’s HSD Test using computer software Statistix 8.1.

### Ethics approval

This article does not contain any studies with human participants or animal experiments.
